# Retraction: Epiploic Appendagitis Clinically Masquerading as an Acute Diverticulitis

**DOI:** 10.7759/cureus.r109

**Published:** 2024-01-25

**Authors:** Razan A Khafaji, Hussain S Ghandourah, Sarah K Altamimi, Afnan A Alwarthan, Renda A Alhabib, Mazen N Alaiyar, Ibrahim A Alomar, Meshari I Alayshan, Mohammed S Almasoudi, Hashem A Jaml Allil, Shahad Z Munshi, Sarah K Aljamri, Basil S Bagadeem, Motaz S Attar, Faisal Al-Hawaj

**Affiliations:** 1 College of Medicine, King Abdulaziz University, Jeddah, SAU; 2 College of Medicine, Maastricht University, Maastricht, NLD; 3 College of Medicine, Arabian Gulf University, Manama, BHR; 4 College of Medicine, King Saud bin Abdulaziz University for Health Sciences, Riyadh, SAU; 5 College of Medicine, Qassim University, Qassim, SAU; 6 College of Medicine, AlMaarefa University, Riyadh, SAU; 7 College of Medicine, Jouf University, Al-Jawf, SAU; 8 College of Medicine, Umm Al-Qura University, Mecca, SAU; 9 College of Medicine, Ibn Sina National College for Medical Studies, Jeddah, SAU; 10 College of Medicine, Imam Abdulrahman Bin Faisal University, Dammam, SAU; 11 College of Medicine, King Saud Bin Abdulaziz University for Health Sciences, Jeddah, SAU

The Editors-in-Chief have retracted this article. Concerns were raised regarding the identity of the authors on this article. Specifically, Faisal Alhaway and Malak Shammari have stated that they were added to this article without their knowledge or approval. The identity of the other authors could also not be verified. As the appropriate authorship of this work cannot be established, the Editors-in-Chief no longer have confidence in the results and conclusions of this article.

